# A prenatal group based phone counseling intervention to improve breastfeeding rates and complementary feeding: a randomized, controlled pilot and feasibility trial

**DOI:** 10.1186/s12884-021-03976-2

**Published:** 2021-07-22

**Authors:** Jennifer S. Cauble, Amy Herman, Jo Wick, Jeannine Goetz, Christine M. Daley, Debra K. Sullivan, Holly R. Hull

**Affiliations:** 1 School of Health Professions, Department of Dietetics & Nutrition, University of Kansas Medical Center, 3901 Rainbow BLVD, Mail Stop 4013, Kansas City, KS 66160 USA; 2 School of Medicine, Department of Preventative Medicine and Public Health, University of Kansas Medical Center, Kansas City, USA

**Keywords:** Breastfeeding, Introducing Solids, Prenatal Education, Infant Feeding, Lactation, Human milk

## Abstract

**Background:**

Despite numerous benefits for both mom and baby, few infants are exclusively breastfed for the recommended first six months. Additionally, infants are given solids too early. Prenatal education increases rates of breastfeeding initiation and we hypothesize it can also improve exclusive breastfeeding rates and prevent the early introduction of solids. We conducted a randomized controlled pilot and feasibility trial to understand the feasibility and maternal acceptance of a prenatal behavioral lifestyle intervention (PBLI) delivered via group based phone counseling (GBPC) and its effectiveness on rates of exclusive breastfeeding up to six months postpartum. Secondary aims included rates of any breastfeeding up to six months, rates of early introduction of solids, and infant feeding progression.

**Methods:**

Forty-one pregnant women were recruited from a Kansas City Metropolitan Obstetrics and Gynecology office and randomly assigned to a usual care group or a PBLI. Women in the PBLI participated in six GBPC sessions where they learned about breastfeeding and introducing solids. Feeding questionnaires to assess breastfeeding and introduction of solids were sent at two weeks, two months, four months, and six months postpartum. Structured interviews were also conducted after the intervention and at six months postpartum to assess maternal acceptance and intervention feasibility.

**Results:**

Participants overwhelmingly found the intervention acceptable and beneficial.

Rates of exclusive breastfeeding and any breastfeeding did not differ between groups at any time point. No between group differences were found for early introduction of solids or infant feeding progression.

**Conclusions:**

Mothers discontinue breastfeeding earlier than recommended despite high rates of initiation. A PBLI delivered via GBP is feasible, acceptable to participants, and showed positive impacts such as maternal empowerment for both breastfeeding and introducing solids. Future interventions should incorporate both prenatal and postpartum components.

**Trial registration:**

Study protocols were approved by the University of Kansas Medical Center’s Human Subjects Committee (STUDY00140506) and registered at ClinicalTrials.gov on 02/22/2018 (NCT03442517, retrospectively registered). All participants gave written informed consent prior to data collection.

## Background

Meeting infant feeding recommendations is a public health priority due to the numerous benefits for both mom and infant. This includes both duration and exclusivity of breastfeeding and preventing early introduction of solids. The benefits of breastfeeding for both mom and baby are well established [1]. Introducing solid foods prior to four months is related to childhood obesity development [2] as well as eczema [3], celiac disease [4], and Type 1 diabetes [5, 6]. In addition to suboptimal health outcomes, not meeting infant feeding recommendations costs the US $13 billion annually in pediatric medical costs by contributing to the development of childhood obesity and other diseases [7]. Exclusive breastfeeding is recommended by the American Academy of Pediatrics (APP) for about the about six months, at which time solids can be introduced, with continued breastfeeding to 12 months or longer [1]. Current breastfeeding initiation rates in the United States are high at 83.2%, but by three months of age, only 46.9% of infants are still exclusively breastfeeding and at six months only 24.9% are still exclusively breastfeeding [8]. About 40.4% of infants are currently receiving solids before four months of age [9].

Despite current interventions to improve breastfeeding rates and reduce the early introduction of solids, breastfeeding rates remain low and infants are given solids too early [8, 9]. A review of interventions to improve breastfeeding rates [10] and the timing of solid food introduction [11] reveals that the majority of interventions occur in the postpartum period, but have variable success rates. Addressing barriers and concerns in the postpartum period may be too late. Mothers face new barriers in the postpartum period such as limited time, prioritizing other familial needs, and poor support from family, friends, or coworkers [12]. A lack of prenatal education is a barrier to improved breastfeeding rates [13, 14]. However, advice from a medical professional and breastfeeding education during the prenatal period is associated with increased breastfeeding rates [15]. Therefore, novel interventions in the prenatal period are needed to reduce barriers so mothers receive appropriate infant feeding education and support.

We designed a pilot study to understand if a prenatal behavioral lifestyle intervention (PBLI) delivered using group based phone counselling (GBPC) would be feasible and acceptable to participants and also impact rates of exclusive breastfeeding and any breastfeeding at two weeks, two months, four months, and six months. Secondary aims were to understand if the intervention would impact the rates of early introduction of solids and result in differences in infant feeding progression up to six months.

## Methods

### Study design

The present study is a randomized, controlled pilot and feasibility trial.

### Subjects and randomization

Pregnant women, 18–35 years old, who were 9–30 weeks in gestation and pregnant with their first child or who had exclusively breastfed for less than three months with a previous child were recruited from Northland Obstetrics & Gynecology, Inc between January 2018 and May 2018. Participants were not compensated.

**Fig. 1:**
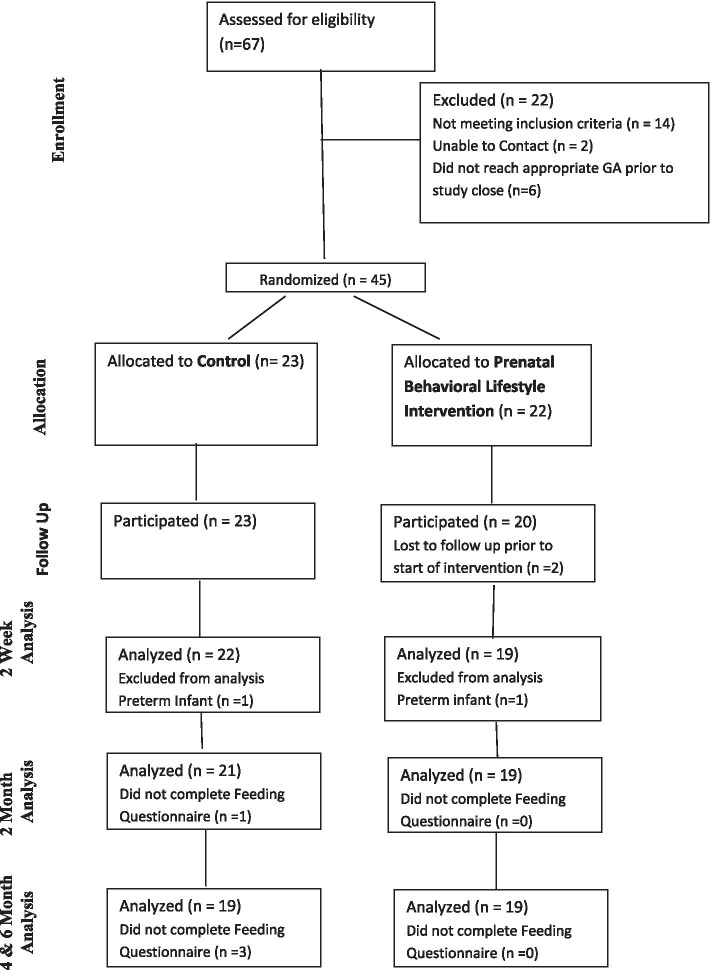
Consort Diagram

Due to the effect on pregnancy and potential complications related to breastfeeding after delivery (i.e. poor milk production), women with pregnancies conceived using fertility treatments, those at high risk for pre-term delivery, those with multiple gestation (i.e.. twins, triplets, etc.), or pregnancies complicated by morbid obesity (BMI > 40), diabetes (pre-gestational or gestational), hypertension, metabolic dysfunction, etc., were excluded. Women who developed any of these conditions during pregnancy or had a preterm infant (< 37 weeks) were excluded from the final analysis. A CONSORT diagram is included in Fig. 1. Participants were block randomized in groups of 6–10 into either the PBLI intervention or usual care group at a 1:1 ratio. The allocation sequence was computer-generated by the study statistician and given to the PI. After completion of enrollment, the PI provided the participant’s allocation to the study team member: usual care or the intervention group. If women indicated to their provider they were interested in hearing about a breastfeeding study, they were approached at their regularly scheduled obstetrician and gynecologist (OBGYN) clinic appointment. A research team member discussed the study with the women while still in clinic. If unavailable to meet in clinic, women were provided a study flyer, and with consent of each individual patient, the OBGYN office provided the contact information to research staff. Research staff called women to discuss study participation. Once women indicated interest, they were screened for eligibility. Eligible women that agreed to participate were consented in person or via phone using a REDCap **(REDCap, RRID:SCR_003445)** [16, 17] link. The study statistician was blinded for randomization but the study participants and study team members were not.

### Prenatal behavioral lifestyle intervention

Intervention participants participated in six weekly GBPC sessions starting between 16–30 weeks gestation. Three intervention groups containing between 6–10 participants each were held. Phone calls were conducted using the Acano Audio Conferencing System. Each session was approximately 60 min and was led by an International Board Certified Lactation Consultant (IBCLC) and registered dietitian (JSC). Participants were given a comprehensive manual that outlined weekly lessons including Introduction to Breastfeeding, Breastfeeding Basics, Pumping 101, Back to Work, Introducing Solids, and Nutrition and Physical Activity for Breastfeeding. The lessons were didactic in nature, but each lesson encouraged group participation by incorporating participant questions, discussion, and assigned tasks for the next week. Table 1 depicts lesson title and information covered within each lesson.

**Table 1:** PBLI Lessons

Lesson	Objectives of Lesson
Introduction to Breastfeeding	Understand what to feed baby Understand the benefits of breastfeeding Learn the recommendations for breastfeeding duration Compare breastfeeding myths
Breastfeeding Basics	Understand the parts of the breast Learn how breastmilk is made in your body Learn about supply and demand Learn how much baby should be eating Learn common breastfeeding difficulties and solutions
Pumping 101	Understand why pumping is useful Learn how to stare, thaw, and warm milk Understand how to start pumping Learn about different pumps Understand how to increase supply
Back to work	Understand legal rights at work Understand how to prepare for return to work Learn about common breastfeeding difficulties once back to work
Complementary Feeding	Understand when to start solids Learn what type of solid foods to feed your baby Understand how much your infant should be eating Explore mealtime tips and choking hazards
Nutrition and Physical Activity for Breastfeeding	Understand what to eat while breastfeeding Understand nutrient density Learn when to incorporate physical activity Learn practical tips for eating healthy after baby

### Usual care

Participants in the usual care group received standard pregnancy and pediatric education provided by their healthcare provider. They received no additional breastfeeding or nutrition education.

### Data collection

#### Demographic questionnaire

A data collection timeline is presented in Table 2. Women were emailed a REDCap (REDCap, RRID:SCR_003445) questionnaire to collect demographic data [16, 17] at the time of study enrollment. The questionnaire collected data including height, pre-pregnancy weight, age, sociodemographic information (income, education, and employment status), and previous number of pregnancies.

**Table 2:** Enrollment and Intervention Timeline

Baseline (9-30wks gestation)	Intervention (16-30wks gestation)	Post- Intervention	1 week	2 week	2 month	4 month	6 month
Baseline Characteristics Questionnaire							
	6 weekly GBPC calls						
			Feeding survey	Feeding survey	Feeding survey	Feeding survey	Feeding survey
		Structured Interview (intervention only)					Structured Interview (intervention only)

### Breastfeeding and introduction of solids

At two weeks, two months, four months, and six months postpartum, women were sent a REDCap (REDCap, RRID:SCR_003445) [16, 17] survey. Women answered questions regarding breastfeeding, use of formula, and introduction or use of solid foods. This information was used to classify breastfeeding status and to assess timing of solid food introduction. For infants less than four months of age, exclusive breastfeeding was defined using the World Health Organization (WHO) guidelines, which state that exclusively breastfed infants only receive human milk. No other liquids or solids are given, not even water, with the exception of oral rehydration solutions, drops/syrups, minerals, or medicine [18]. We altered our definition of exclusive breastfeeding after four months to encompass infants being provided human milk only (no formula) but also receiving solid foods. This was based on current recommendations from the AAP stating that in combination with providing only human milk, solid foods can begin between four and six months of age, with developmental readiness as a guide [19]. If women did not meet the criteria for “exclusive breastfeeding” but were offering human milk to some extent, they were classified as “any breastfeeding.”

#### Structured interviews

Immediately following intervention completion and at six months postpartum, a structured interview was completed by those who participated in the intervention to understand maternal acceptance of the intervention including benefits and potential improvements. Structured interview questions are displayed in Table 3.

**Table 3:** Structured Interview Questions

Structured Interview Questions
**Post Intervention Questions**
*Overall*
What did you like about this program?
What did you dislike about this program?
Did the intervention help you determine how you wanted to feed your baby?
*Calls*
Were the weekly group calls at a time you were generally available?
What did you like about the phone meetings and what did you not like about the phone meetings?
Was there anything about the calls that you would change?
Would you participate in another intervention using phone meetings?
*Future*
What do you think we could do in order to make this program better?
Would you recommend this program to a friend?
For the future, instead of a phone meeting would you like to receive information in a different way such as a short video format, manual only, in person, etc.?
6 Month Follow up Questions:
*Overall*
Do you feel the information you received on the phone calls was beneficial for breastfeeding your baby and introducing solids, if you have done that yet?
What information that you received on the phone calls did you find most helpful while breastfeeding and introducing solids, if you have done that yet?
Is there any information you did not receive during the phone calls that you wish you had received that would have made breastfeeding or introducing solids more successful?
Did you use your participant handbook after baby arrived to look up information?
Do you feel participating in the intervention helped you reach your breastfeeding goals? Any overall feedback that you would like to give?

### Data analysis

Frequencies and proportions were calculated for all categorical variables. Means and standard deviations were calculated for all continuous variables. Continuous variables were checked for normality. Exact binomial confidence intervals were calculated for rates of exclusive breastfeeding and any breastfeeding, and ${x}^{2}$ or Fisher’s exact tests were used to compare rates between groups. Mean duration and 95% CI of exclusive breastfeeding and introduction of solids were obtained via Kaplan–Meier survival curves. An intent-to-treat analysis was conducted, including any subjects that did not participate in the GBPC sessions. A secondary analysis included those considered compliant to the protocol, attending at least four of the GBPC sessions. Data were analyzed using SPSS **(IBM SPSS Statistics, RRID:SCR_019096)** version 25.0 and SAS 9.4 with a p-value ≤ 0.05 considered statistically significant. Structured interviews were recorded using the Acano Audio Conferencing System. A thematic analysis was completed. Verbatim transcripts were created using Temi Transcription service (Temi.com, San Francisco, CA). The research coordinator coded the transcripts and identified topics within each question to create a key. Three individual study personnel (the research coordinator (JSC), research assistant (AH), and PI (HH)), used the transcripts and the key to deductively abstract the data into topics. Each team member inductively coded the transcripts. All coders identified preliminary themes which were sent to an outside researcher (CMD) to develop thematic statements. All illustrative quotes were identified by the moderator/research coordinator (JSC).

## Results

### Demographics

Sixty-seven women were screened, and 53 were eligible (Fig. 1). The primary reason for exclusion was previous breastfeeding experience (7%) or elevated BMI (6%). Of the eligible women (n = 53), 45 women consented, for an enrollment rate of 85%. Twenty-three women were randomized to the usual care group and 22 were randomized to the intervention. Prior to the start of the intervention, two women in the intervention group were lost to follow up. Post-delivery, one participant in the usual care group and one participant in the intervention group were excluded due to a preterm delivery. Overall, 41 women (usual care n = 22 and intervention n = 19) were used for analysis. For the usual care group, all women completed the two-week questionnaire, one woman did not complete the two month, four month, and six month feeding questionnaires and two women did not complete the four and six month feeding questionnaires. For the intervention group, all women completed all feeding questionnaires at all time points.

No between group differences were found for the baseline characteristics (Table 4). The mean participant age was 26 years (SD: 4.3 years) with an average BMI of 27.3 kg/m^2^ (SD: 4.5). Most women were white (95.1%) and 65.9% had an Associate’s degree or higher. Women were primarily married or co-habitating with their significant other (82.9%) with 46.3% having a household income less than $75,000 per year. Most women were having their first child (70.7%). Infants were primarily born via vaginal delivery (84.2%). The mean gestational age at birth was 39.5 weeks (SD: 1 week) and mean birthweight was 7.8 lbs (SD: 1.0 lbs). Infant gender was evenly split with 48.8% of infants being female.

**Table 4:** Maternal and Infant Characteristics

	Overall *N* = 41	Usual care *N* = 22	Intervention *N* = 19	*P*-Value
Maternal				
Age (years)	26.2 ± 4.3	25.4 ± 4.5	27.3 ± 4.1	0.1
Pre-pregnancy BMI (kg/m^2^)	27.3 ± 4.5	26.8 ± 4.4	27.9 ± 4.7	0.4
White Race n(%)	39 (95.1%)	21 (95.5%)	18 (94.7%)	1.0^¥^
Education n(%)				0.9^¥^
Less than High School	1 (2.4%)	1 (4.5%)	0 (0%)	
GED	3 (7.3%)	2 (9.1%)	1 (5.3%)	
High School	9 (22%)	4 (18.2%)	5 (26.3%)	
Vocational	1 (2.4%)	1 (4.5%)	0 (0%)	
Associates Degree	4 (9.8%)	3 (13.6%)	1 (5.3%)	
Undergraduate Degree	16 (39%)	8 (36.4%)	8 (42.1%)	
Graduate Degree	7 (17.1%)	3 (13.6%)	4 (21.1%)	
Married or Cohabitating n(%)	34 (82.9%)	17 (77.3%)	17 (89.5%)	0.4^¥^
Household Income n(%)				0.9
≤ $75,000	19 (46.3%)	10 (45.5%)	9 (47.4%)	
> $75,000	22 (53.7%)	12 (54.5%)	10 (52.6%)	
Parity, Primiparous n(%)	29 (70.7%)	15 (68.2%)	14 (73.7%)	0.7
Type of Delivery n(%)				1.0^¥^
Vaginal	32 (84.2%)	16 (84.2%)	16 (84.2%)	
Cesarean	6 (15.8%)	3 (15.8%)	3 (15.8%)	
Infant				
Gestational age (weeks)	39.47 ± 1.00	39.49 ± 0.78	39.46 ± 1.24	0.9
Female n(%)	30 (48.8%)	11 (50%)	9 (47.4%)	0.8
Birthweight (lbs)	7.80 ± 1.03	7.69 ± 1.00	7.93 ± 1.09	0.4

Values are % or mean ± SD¥: Fisher’s exact test

### Lactation support

Most women (usual care = 94.7%, intervention = 73.7%) received lactation support in the hospital after delivery. Post-discharge, 47.7% of the women in the usual care group received lactation support compared to 73.7% of women in the intervention. Women primarily received post-discharge lactation support from a lactation professional (usual care = 36.8%, intervention = 63.2%) but also received support from their OBGYN (usual care = 5.3%, intervention = 5.3%) and the Women Infants and Children (WIC) program (usual care = 0%, intervention = 10.5%).

### Intervention compliance

Intervention compliance was defined as attending a minimum of four phone meetings. Eighty-five percent of the sample (n = 16) attended four or more phone meetings. Only three women did not attend the minimum of four phone meetings. Missed sessions were primarily due to work commitments or appointments that interfered with the session time.

### Rates of breastfeeding initiation, exclusive breastfeeding, and any breastfeeding

Rates of breastfeeding initiation, exclusive breastfeeding, and any breastfeeding at two weeks, two months, four months and six months are displayed in Table 5. All women in the usual care group initiated breastfeeding, while all but one of the women in the intervention initiated breastfeeding showing no between group differences. Next, exlusive breastfeeding rates will be discussed. At 2 weeks, 59.1% of women in the usual care were exclusively breastfeeding compared to 63.2% in the intervention (p = 0.54). At two months, 50% of women in the usual care group were exclusively breastfeeding compared to 52.6% in the intervention (p = 0.7). At four months, rates were 31.8% in the usual care compared to 31.6% in the intervention (p = 0.97). Finally, rates of exclusive breastfeeding at six months were similar in the intervention group (31.6%) and the usual care group (31.8%) with a p value = 0.97. No between group difference was found for exclusive breastfeeding at any time point. Exclusive breastfeeding rates declined until four months and then remained stable at six months in the usual care group and intervention group. The largest drop in exclusive breastfeeding for both groups occurred after two months.

**Table 5:** Rates of Initiation, Exclusive Breastfeeding, and Any Breastfeeding

	Usual care *n* = 22	Intervention *n* = 19	*P*-Value Usual care vs Intervention	Protocol Compliant *n* = 16	*P*-Value Usual care vs protocol compliant
BF Initiation					
	22 (100%)	18 (94.7%)	0.5 ¥	16 (100%)	-
Exclusive Breastfeeding					
2 weeks	13 (59.1%)	12 (63.2%)	0.79	11 (68.8%)	0.54
2 Months	11 (50%)	10 (52.6%)	0.87	9 (56.3%)	0.7
4 Months	7 (31.8%)	6 (31.6%)	0.98	5 (31.3%)	0.97
6 Months	7 (31.8%)	6 (31.6%)	0.98	5 (31.3%)	0.97
Any Breastfeeding					
2 Weeks	19 (86.4%)	17 (89.5%)	0.35¥	16 (100%)	0.25¥
2 Months	13 (61.9%)	14 (73.7%)	0.43	13 (81.3%)	0.28¥
4 Months	10 (52.6%)	11 (57.9%)	0.74	10 (62.5%)	0.56
6 Months	9 (47.4%)	9 (47.4%)	1.0	8 (50%)	0.88

Values are n(%)¥: Fisher’s exact test

Next, the results related to any breastfeeding will be discussed. At two weeks, 86.4% of women in the usual care group were participating in any breastfeeding compared to 89.5% in the intervention (p = 0.25). At two months, 61.9% of women in the usual care were still breastfeeding compared to 73.7% in the intervention group (p = 0.28). At four months, rates were 52.6% in the usual group compared to 57.9% in the intervention (p = 0.56). Finally, rates of any breastfeeding at six months in the usual care group were 47.7% and 47.7% in the intervention (p = 0.88). No between group difference was found for any breastfeeding at any time point. Overall, as the infant aged, breastfeeding rates declined at each successive time period in the usual care group and intervention.

In a secondary analysis of women compliant to the intervention compared to usual care, we found that all women initiated breastfeeding. No difference was found for rates of any breastfeeding or exclusive breastfeeding at any time point. Exclusive breastfeeding rates also declined at two weeks, two months, and four months, but then remained stable from four months to six months.

### Reasons for formula introduction

During the first six months, women were asked to identify if they had introduced formula and reasons for formula introduction (Table 6). At two weeks the main response was “other” (n = 8) and “following advice from a healthcare provider” (n = 6). The most common listed reasons for “other” were milk supply (n = 3) and latching issues (n = 2). At two months, the primary answer was “other” (n = 9) and “baby did not gain enough weight on breastmilk alone” (n = 6). Listed “other” reasons were all related to milk supply problems (n = 5) or poor support (n = 1) with three women giving no response. At four months, the primary reason for formula introduction was “other” (n = 14) and “easier to fit into daily routine.” The main listed reason under “other” was milk supply (n = 8). At six months, the primary reason for formula introduction was “other” (n = 10) and “easier to fit into daily routine” (n = 9). The main listed reason under “other” was milk supply (n = 7).

**Table 6:** Reasons for Introduction of Formula. Data are reported as n(%)

	2 Weeks n = 16	2 Months n = 20	4 Months n = 25	6 Months n = 25
Following Advice from HealthCare Provider	6 (37.5%)	4 (20.0%)	5 (20.0%)	5 (20.0%)
Following Advice from Family and Friends	1 (6.3%)	0 (0.0%)	0 (0.0%)	0 (0.0%)
Breastfeeding was too Difficult	3 (18.8%)	3 (15.0%)	3 (12.0%)	4 (16.0%)
Baby Did Not Gain Enough Weight	4 (25.0%)	6 (30.0%)	5 (20.0%)	5 (20.0%)
Easier to Fit into Daily Routine	2 (12.5%)	7 (35.0%)	8 (32.0%)	9 (36.0%)
Allows Others to Feed Baby	2 (12.5%)	4 (20.0%)	6 (24.0%)	4 (16.0%)
My Plan was to Formula Feed	1 (6.3%)	1 (5.0%)	1 (4.0%)	1 (4.0%)
Other	8 (50.0%)	9 (40.0%)	16 (60.0%)	9 (36.0%)
	Supply (3) Latch (2) Mental Health (1) BF isn’t always possible (1) No answer (1)	Supply (5) Poor Support (1) No answer (3)	Supply (8) Mental Health (2) No Answer (2) Refused to BF (1) Poor Support (1)	Supply (7) Mental Health (2) Refuse to BF/Weight loss (1)

### Introducing solids

No between group difference was found for the timing of solid introduction. Most infants in both the usual care group (94.7%, 95% CI = 74%-99%; n = 18) and intervention group (94.7%, 95% CI = 74%-99%; n = 18) started solid foods appropriately and no infant received solids at or before two months. One infant in both the usual care group and intervention group started solids prior to four months of age. The remaining infants in the usual care group and intervention were given solids after four months. When asked to mark all options that influenced their decision to start solid foods, 28 women (usual care n = 13, intervention n = 15) indicated “baby was showing interest,” 17 women (usual care n = 6, intervention n = 11) indicated they were “following the advice of a healthcare provider,” six women (usual care n = 3, intervention n = 3) indicated that “breastmilk or formula alone was not enough,” one woman in the intervention indicated she was “following advice from family or friends,” and one woman in the usual care group indicated “other” but did not specify her reason.

### Feeding progression

**Fig. 2:**
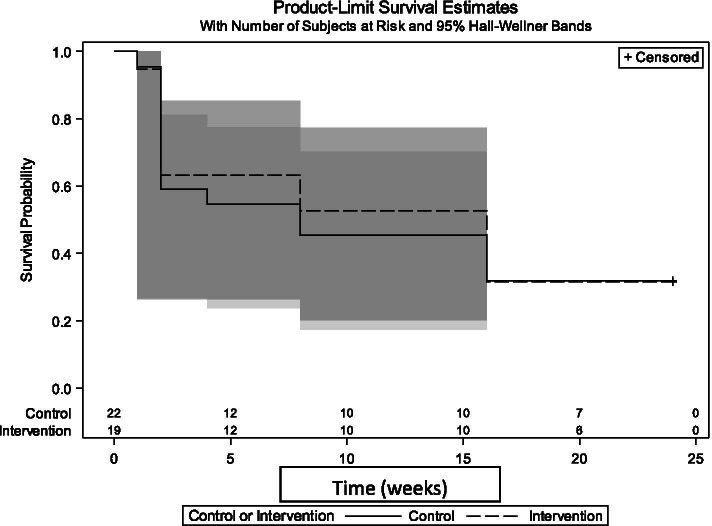
Overall duration of exclusive breastfeeding by treatment group

Figure 2 presents the overall time until the cessation of exclusive breastfeeding over the six-month follow up period for each group. There were no between group differences for rates of exclusive breastfeeding at any time points (log-rank *p* = 0.87). Overall, exclusive breastfeeding dropped dramatically between birth and two weeks and then continued to decline until six months. In the usual care group, the mean age for discontinuation of exclusive breastfeeding was nine weeks (SD ± 1.5 weeks) versus 10 weeks (SD ± 1.6 weeks) in the intervention group. Seven women in the usual care group and six women in the intervention group were still exclusively breastfeeding at six months.

**Fig. 3:**
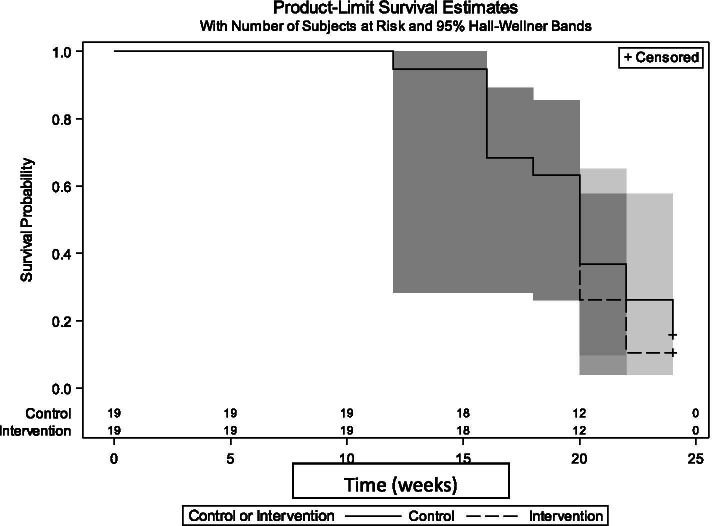
Overall time until introduction of solids by treatment group

Figure 3 presents the overall time to introduction of solids between groups. No differences between groups were found regarding the time for introduction of solids (log-rank *p* = 0.57). One infant in both the usual care and intervention started solids early, prior to four months. The mean age for introduction of solids in the usual care group was 4.9 months (SD ± 0.75 months) and 4.7 months (SD ± 0.65 months) in the intervention. At six months, three infants in the usual care group and two infants in the intervention group had not started solids.

### Maternal perception of intervention

#### Post intervention

Four primary themes emerged from the thematic analysis of the structured interview responses immediately after the intervention concluded. The first theme was that women liked the program including the format, accompanying manual, the diversity of experiences represented from group members, having an expert available for discussion, and the comprehensiveness of the information received. They also mentioned several other positive factors such as having the information broken into sections, not having to travel anywhere for group meetings, and having an hour set aside to focus on learning about the topic, to name a few. Overall, women felt the amount of information provided was appropriate and were positive about their GBPC experience. The second theme was that women would participate in another intervention delivered via GBPC. When asked about participating in another PBLI delivered with GBPC, one woman stated that the intervention “made me feel more empowered as a woman who is going into taking care of their first child. I feel like I have more tools, and it’s crazy because it’s just a talking session, but you know, knowledge is power when it comes down to it.” The third theme was that the intervention helped women decide how they wanted to feed their infant and/or supported the feeding decision they had already made. Some women indicated they had already decided how they wanted to feed their baby prior to the intervention. One woman stated, “the program solidified what I wanted to do, because I already had that plan in mind (to breastfeed) but, this gave me a roadmap of how to do it.” The final theme was that women were positive about their GBPC experience but provided constructive feedback. One mom stated, “I loved the way it was set up.” Another said, “I thought it was super informative.” Two primary concerns were lack of connectivity and engagement, which in turn made conversation more difficult for some women. Women indicated they wanted at least one in-person meeting to build rapport with group members. Women also wanted additional visuals to augment the phone calls such as videos or web links and to have calls recorded so they could listen to them later.

#### Six month follow-up

Thematic analysis of the structured interviews conducted at six months postpartum revealed five themes which included 1) Women retained a positive perception of the intervention after having a baby and starting their infant feeding journey 2) Women used their participant manuals after the baby arrived 3) Women had suggestions on program improvement 4) Women wished the program encompassed both the prenatal and postpartum period 5) Women that in the intervention who did not meet their goals wished there was additional support in the postpartum period. The first theme was that, women retained a positive perception of the intervention after having a baby and starting their infant feeding journey. The intervention was particularly helpful for breastfeeding, but also for introducing solids. One woman stated, “It gave me the confidence to get started on the right foot.” Another mom stated, “I really felt comfortable going into breastfeeding.” Several women had not started solid foods yet to give adequate feedback on how the intervention helped or needed improvement. The second theme was that women used their participant manuals after the baby arrived. Regarding the use of the manual postpartum, one mom stated, “If I was having an issue with latching or if I was having a problem I knew exactly where to find it and it was super simplified, and it told me exactly what I needed to know.” The last three themes discussed potential improvements to the program. The third theme was that after the women had the chance to implement the information they learned, they had some specific suggestions on information they felt would benefit the program, particularly regarding breastfeeding. This included additional information on initiating breastfeeding in the hospital, breastfeeding and going back to work, latching, pumping, the use of nipple shields, and supply problems. The fourth theme was that women, regardless of if they were successful at breastfeeding or not, indicated they wished the program had encompassed both the prenatal and postpartum time period, so sessions and group support continued after the baby arrived. One woman stated, “it should continue on to when you are actually doing (breastfeeding and introducing solids) so that you can get real time advice and feedback.” The final theme was from women who were not able to meet their goals. They felt the intervention was helpful but wished there was additional support in the postpartum period. One woman stated “The phone calls helped but they were not (enough). I needed someone in the room to help guide me.”

## Discussion

This study demonstrated the feasibility and acceptability of a PBLI to be delivered via GBCP as a potential method to improve breastfeeding rates and the timing for introducing solids. Overall, the study found no difference in breastfeeding rates (initiation, any breastfeeding, or exclusive breastfeeding) at any time point. There was no difference in rates of early introduction of solids or feeding progression between groups. As a pilot project, this study was not powered to detect statistical differences between groups. As such, it is not surprising that no significant differences were found. Despite the lack of statistical difference, the rates of breastfeeding that we found can be used to help inform future interventions. Overall, the intervention was found to be feasible, acceptable, and beneficial by participants.

### Maternal perception of the intervention

Despite no difference in rates of breastfeeding or early introduction of solids, women indicated the program either helped them decide how to feed their baby or supported the decision they had already made. The intervention also made them feel more prepared and confident.

Women offered constructive suggestions to improve future interventions including additional information on breastfeeding in the hospital, going back to work, latching, increasing supply, nipple shield use, and pumping. These suggestions support our findings that primary reasons for formula introduction were milk supply and latching concerns. Women also wanted more information on introducing solids as it was only discussed in one lesson. Specific feedback regarding what women liked and disliked about the introducing solids information was limited as several women had yet to utilize the information. Some women felt the introducing solids lesson was offered too soon as it was still too far in the future for the information to be relatable. Women consistently commented on having access to additional help for both breastfeeding and introducing solids in the postpartum period. Future studies should address maternal suggestions to further refine the intervention.

### Intervention delivery method

To our knowledge, this is the first prenatal breastfeeding intervention delivered via GBPC. Previous studies found that traditional face to face interventions are effective, but they also present a higher cost and an increased number of barriers for participants [20]. Previous studies found delivering intervention information via technology to be feasible and effective [21]. While GBPC to improve breastfeeding rates has not previously been utilized in pregnancy, it has been used in adult weight management and shown equivalent levels of weight loss when compared to traditional face-to-face methods [22].

For the present study, 85% (n = 16) of the sample was compliant and the PBLI had high acceptability. In the usual care group, there was a 100% response rate at two weeks, 95% response rate at two months, and 86.3% response rate at both four and six months. The intervention group response rate was 100% at all time points. Women liked that the intervention was delivered remotely (including the remote delivery of surveys) allowing them to stay home and avoid travel concerns, the overall information that was provided, the structure of the program (including the comprehensive manual and homework) and being on the phone with a group of women going through a similar life stage. When asked, all women indicated they would participate in an intervention delivered via GBPC again. Additionally, they felt the GBPC was the optimal method of delivery for the intervention; however, there were concerns about a lack of engagement and connectivity and some women desired additional visuals such as video links. Women proposed options such as a single in-person meeting prior to the start of the intervention or a method such as video chat to improve rapport between women in the group and thus engagement.

### Breastfeeding

Our results are similar to a study by Schreck et al. [23] who found a prenatal intervention alone was ineffective at improving breastfeeding continuation. Attendance at a postpartum support group was required to see higher rates of continuation. Despite women having positive feeling about receiving education in the prenatal period, our results confirm that women need and want additional support in the postpartum period in conjunction with prenatal education. Women who struggled to meet their breastfeeding goals wanted postpartum meetings or access to “real time” advice after the baby arrived to help troubleshoot specific concerns. We know that postpartum support groups are beneficial for increasing breastfeeding rates as they allow for women to address issues in “real time” as problems arise [24, 25]. To our knowledge, a postpartum GBPC intervention has never be evaluated but would provide for “real time” advice that women desire. A future intervention to improve breastfeeding rates that incorporates group based phone counseling across the span of both the prenatal and postpartum period that is adequately powered is warranted.

### Introduction of solids

This is the first randomized controlled trial to examine the effect of an educational intervention delivered in the prenatal period on rates of early introduction of solids (prior to four months). No between group difference was found for the timing of solid introduction. In both the usual care and intervention, only one infant in each group received solids before four months. These rates are surprisingly low, accounting for only 5% of the group. Previous research indicates rates of early introduction of solids to be 24.3% in exclusively breastfed infants, 50.2% in mixed-fed infants, and 52.7% in exclusively formula fed infants [9]. These results may be explained in part by the characteristics of our sample. According to Hendricks et al. [26] introducing solids prior to four months is associated with younger maternal age, being African American, living in a household below 185% federal poverty level, and having less than a college education. Our sample consisted of women in their late twenties (mean 26 years old SD: 4.3 years), white, educated, and 53.7% reporting a household income above $75,000.

In summary, GBPC, and more specifically the program format and content used for this study was an effective and acceptable method for intervention delivery and should be considered in future studies. Future studies should adjust the curriculum as suggested by participants and specifically add additional information on proper latch and maintaining an adequate milk supply throughout breastfeeding. An additional postpartum group component should be considered as this may be a vital component to improving breastfeeding rates. Finally, an effective technological method for improving breastfeeding rates and preventing the early introduction of solids that reduces both financial and participant barriers could drastically increase the number of women who received appropriate infant feeding information and improve infant and maternal health outcomes.

#### Strengths and limitations

A strength is that an evaluation of maternal satisfaction of the intervention was completed to help guide future intervention development. This component was previously underreported in research [27]. Another strength is the high response rate and compliance to the study protocol. However, our study also had some limitations. For this study, we used maternal self-report for breastfeeding outcomes; however, previous research has shown this to be a reliable measure [28]. Another limitation is the relative homogeneity of the women in both the intervention group and the control group limiting generalizability. We do not know how women in other populations would respond to the intervention. Further, women in both groups received lactation help in both the hospital and after discharge. We do not know what affect this may have had on their decision to continue breastfeeding. Another limitation is the small sample size and lack of power to detect significant results. A future study, with similar design, that is adequately powered is needed to determine the effect of a PBLI intervention delivered via GBPC on breastfeeding rates and introduction of solids.

## Conclusions

Despite high initiation rates, women are discontinuing breastfeeding before recommended. Women overwhelmingly found the intervention beneficial and felt it gave them confidence and prepared them to breastfeed and introduce solids to their infant. Results from our pilot and feasibility study found that GBPC is an acceptable method of delivering a PBLI intervention for educating women on appropriate infant feeding. The intervention was not powered to detect statistically different results for breastfeeding rates. In the future, a larger, adequately powered study delivered via GBPC should be evaluated with a combination of prenatal education and postpartum support.

## Data Availability

The datasets used and/or analyzed during the current study are available from the corresponding author on reasonable request. If an investigator would like to collaborate with the study team to complete an analysis and prepare a manuscript, an abstract detailing the research question, background to support gap in literature, statistical analyses to be completed, a timeline for completion of project, and three journals for planned submission should be sent to the corresponding author for consideration. All requests will be considered in the order they are received.

## Abbreviations

AAP: American Academy of Pediatrics
PBLI: Prenatal Behavioral Lifestyle Intervention
GBPC: Group Based Phone Counseling
IBCLC: International Board Certified Lactation Consultant
WHO: World Health Organization
